# Prevalence, sociodemographic factors, psychological distress, and coping strategies related to compulsive buying: a cross sectional study in Galicia, Spain

**DOI:** 10.1186/1471-244X-14-101

**Published:** 2014-04-05

**Authors:** José Manuel Otero-López, Estíbaliz Villardefrancos

**Affiliations:** 1 Department of Clinical Psychology and Psychobiology, Faculty of Psychology, Campus Vida, University of Santiago de Compostela, Santiago de Compostela 15782, Spain; 2 Faculty of Psychology, Campus Vida, University of Santiago de Compostela, Santiago de Compostela 15782, Spain

**Keywords:** Compulsive buying prevalence, Sociodemographic factors, Psychological distress, Coping strategies

## Abstract

**Background:**

Compulsive buying has become a serious problem affecting a growing number of people in contemporary consumer societies. Nevertheless, research examining its prevalence in representative samples from the general population is still scarce and mainly focused on the exploration of sociodemographic factors, neglecting other aspects like psychological distress and coping styles. Therefore, this study intends to contribute to the cumulative knowledge by assessing compulsive buying prevalence in a representative sample from the general population in the region of Galicia, in Spain. Sociodemographic determinants, psychological symptoms, and coping strategies are also analyzed to clarify their role in this phenomenon.

**Methods:**

A random routes procedure was employed in the recruitment of the sample which was comprised of 2159 participants who were classified as either compulsive buyers or non-compulsive buyers. Both groups were compared regarding sociodemographic determinants, symptoms, and coping strategies through chi-square tests or analyses of variance. A multivariate logistic regression analysis was conducted to determine which of these determinants might play a part in the make up of a risk profile for compulsive buying.

**Results:**

Estimated prevalence of compulsive buying was 7.1%. Compulsive buyers and non-compulsive buyers differed significantly in sex and age, with women and younger people showing a higher propensity for this phenomenon. Individuals with compulsive buying presented significantly higher scores on all the psychological symptoms considered. They also employed passive-avoidance coping strategies much more frequently and active strategies of problem solving and cognitive restructuring much less frequently. The logistic regression analysis results confirmed that being female, experiencing symptoms of anxiety, depression, and obsession-compulsion, and employing the passive-avoidance coping strategies of problem avoidance, wishful thinking, and self-criticism, all constituted risk factors for compulsive buying, whilst the increased age and the use of the active coping strategies of problem solving and cognitive restructuring were protection factors.

**Conclusions:**

Our findings revealed a substantial prevalence of compulsive buying. Additionally, the relevance of sociodemographic determinants, psychological distress, and coping strategies in this problem was confirmed. The establishment of a risk profile for compulsive buying based on these different sets of determinants would likely contribute to the development of more effective intervention programs.

## Background

Compulsive buying has been conceptualized as a chronic and repetitive purchasing pattern which turns into a primary response to negative events or feelings that provides short-term positive rewards, but which ultimately carries harmful consequences [1]. Operational criteria for its study in research have been developed [2,3]. An example being proposed by McElroy et al. [2] who described this behavioural problem in terms of frequent preoccupations with buying, impulses to buy that are experienced as irresistible, and/or frequent shopping episodes in which the person buys more than one can afford, acquires items that are not needed, or invests long periods of time leading to important social and occupational difficulties.

There is a large amount of literature suggesting that compulsive buying constitutes a serious problem [4,5] with growing prevalence in modern consumer societies [6]. In estimating its prevalence, epidemiological surveys on compulsive buying have confirmed percentages ranging from 3.6% [7] to 31.9% [8]. This variability in previous findings with regards to the prevalence of compulsive buying might be influenced by factors such as the type of sample analyzed (students, general population, clinics, for instance), the distinct size of samples, the socio-cultural context, and the employment of different compulsive buying measures. In addition, it should be noted that almost the entirety of the cumulative knowledge in this area has been developed from unrepresentative samples, with representative samples of the general population being one of the contemporary requirements in this field in order to better assess compulsive buying prevalence. Echoing this necessity, some authors have recently explored the prevalence of compulsive buying in representative samples taken from the general population in distinct countries [6,9,10]. Of special note among these studies is that by Neuner et al. [6], who considered representative population-based samples in examining the compulsive buying prevalence in the eastern and the western regions in Germany that were conducted a decade apart. Specifically, in the first survey conducted in 1991, 1% of the East German population, and 5.1% of the West German population were classified as compulsive buyers; later, in 2001 these authors confirmed that the estimated percentages had increased to 6.5% in the east, and 8% in the west showing compulsive buying patterns. Also, in Germany, and using a general population representative sample as starting point, Mueller et al. [10] confirmed that about 7% of the people in this country were compulsive buyers. From a United States’ representative sample, Koran et al. [9] obtained a lifetime prevalence of 5.8% for compulsive buying. This same percentage of 5.8% compulsive buying prevalence was also obtained in a study employing a representative sample taken from the Danish population [11]. But, in spite of these notable exceptions, the scarcity of research employing representative samples emerges as one of the most important gaps in this area. Consequently, this investigation seeks to address this issue and achieve a rigorous approach towards the study of the nature and scope of the compulsive buying phenomenon by examining its prevalence in a large and representative sample taken from the general population in Spain, specifically, from the region of Galicia.

Previous research on compulsive buying is particularly consistent with regards to its multifactorial etiology with a wide plethora of sociodemographic and psychological variables playing a role in the development of this problem [12]. In relation to sociodemographic determinants, gender has received special attention. Thus, from an historical perspective, the vast majority of studies focusing on the relationships between gender and compulsive buying have demonstrated that women show a higher propensity for this phenomenon than men [1,6,13], but it is also true that, in the last decade, and working with representative samples, there have been some studies that have not revealed significant differences based on gender [9,10]. More research appears necessary in order to further clarify the matter. As for age, there is a widespread consensus in the literature regarding the increased vulnerability of younger people to become compulsive buyers [6,9,10,14]. In reviewing the evidence on the role of other demographic variables (marital status, income level, job situation, and education, for example) in compulsive buying, highly inconsistent findings are confirmed. Specifically, whilst findings taken from various studies have shown that compulsive buyers, relative to non-compulsive buyers, are more often single or divorced [7,14], have significantly lower income levels [9], other studies have not confirmed significant differences between compulsive buyers and non-compulsive buyers in these regards [11]. In view of this lack of agreement across studies, there appears to be an urgent and necessary need to advance in the identification of a sociodemographic risk profile for compulsive buying. Therefore, the second objective in this study is to compare compulsive buyers and non-compulsive buyers in the sample with respect to a range of demographic determinants, namely, gender, age, marital status, education, job situation, and perceived social class.

The exploration of distinct dimensions of psychological distress has also emerged as another relevant topic in the researchers’ agenda in the compulsive buying field, with most investigative efforts concentrating on anxiety and depression and producing solid empirical results. In this regard, there is a large body of research demonstrating that compulsive buyers present, when compared to non-compulsive buyers, significantly higher levels either in depression [7,10,15], or in both depression and anxiety [16-18]. Moreover, some studies have shown that these symptoms could act as possible triggers for compulsive buying episodes [17,19,20]. Obsession-compulsion symptoms have also been explored in relation to compulsive buying, but to a lesser extent, with some preliminary findings having revealed significantly higher scores on obsessive-compulsive symptomatology among individuals with compulsive buying [21]. On the other hand, only timid attempts have been made to elucidate the potential implication of other dimensions of psychological distress like hostility [22] and the physical upsets or somatization in the compulsive buying phenomenon [23]. One example is the recent work by Mueller et al. [23], who detected high values in somatization and hostility in a sample of compulsive buyers. Complementary evidence in this regard has also been seen in some studies exploring mood states in relation to compulsive buying episodes [17,24], specifically, Faber and Christenson [17] confirmed that compulsive buyers referred, with a significantly higher frequency than non-compulsive buyers, feelings of anger both before and during compulsive buying episodes. Hence, given the existing evidence regarding the interrelationships between compulsive buying and psychological distress, it is remarkable that previous research has only focused on just a few number of symptoms, particularly anxiety and depression, whilst often neglecting the exploration of others such as obsession-compulsion, hostility and somatization. Within this framework, the current study sets out to explore the relationships between the compulsive buying phenomenon and a variety of symptoms of distinct nature. Thus, by adopting an integrative perspective in relation to psychological distress, we intend to give adequate coverage not only to the emotional side of the question by means of exploring anxiety and depression, but also to the cognitive aspects including obsession-compulsion, without forgetting either physical or interpersonal factors represented by the somatization and hostility dimensions, respectively. Consequently, the examination of the capability of anxiety, depression, obsession-compulsion, hostility, and somatization to establish significant differences between compulsive buyers and non-compulsive buyers constitutes another important goal in the current study.

Surprisingly, research on compulsive buying has paid scarce attention to one of the constructs with the greatest potential in applied settings: coping styles. The prominent role that the strategies which people employ in facing up to difficult circumstances in their lives has acquired in a large number of studies about psychological health underscores this gap in the compulsive buying field. Coping strategies have been widely explored in different areas akin to this phenomenon that include impulse control disorders such as pathological gambling [25,26], eating disorders [27], and alcoholism [28]. Results have demonstrated that, in general, whilst the active-focused on problem coping strategies constitute protection factors against the development of these phenomena, the passive-avoidance coping styles are linked to the initiation and/or the maintenance of the same. New research then, examining the coping strategies-compulsive buying links, appears to be advisable – the exploration of the differences between compulsive buyers and non-compulsive buyers with respect to distinct active and passive-avoidance coping strategies could help clarify the role of these in the problem under study. Hence, this also constituted a relevant goal in this research.

Finally, something that might harbour some important clues for the understanding of compulsive buying would be the development of research of comprehensive character which includes variables of distinct nature, thus making possible the establishment of a risk profile for this behavioural problem. In this regard, it should be noted that most of the previous studies done on representative samples taken from the general population have been primarily centred on the examination of prevalence rates and sociodemographic determinants [6,9,11], giving less attention to the psychological variables. Therefore, in view of the lack of studies which integrate distinct types of variables in an effort to elucidate the potential role of the same as risk or protection factors in relation to compulsive buying, and given the benefits that this approach would mean for the preventive and interventive levels, this study has as its major goal the identification of a prototypical risk profile for compulsive buying that takes into account sociodemographic determinants, psychological symptoms, and coping strategies.

In summarizing, the current study attempts to add to the literature on compulsive buying by means of examining a large and representative general population sample with the following objectives: (a) to estimate the compulsive buying prevalence; (b) to compare compulsive buyers and non-compulsive buyers according to a variety of sociodemographic characteristics; (c) to determine if compulsive buyers differ significantly from non-compulsive buyers in relation to the dimensions of psychological distress of anxiety, depression, obsession-compulsion, somatization, and hostility; (d) to clarify whether the employment of distinct passive-avoidance and active-focused on the problem coping strategies allow for differentiation at significant levels between compulsive and non-compulsive buyers; and (e) to establish a risk profile for compulsive buying based on the sociodemographic determinants, the psychological symptoms, and the coping strategies explored.

## Methods

### Procedure

This investigation was part of a wide ranging research project studying compulsive buying and its associated sociodemographic and psychological variables among the Galician population (Spain). Sample data was collected between September 2012 and April 2013. In recruiting a representative sample from the Autonomous Community of Galicia, members of the research group, along with hired personnel who collaborated in the field work after a training period, travelled to the different localities in this region. Specifically, four regional areas corresponding to the individual provinces in Galicia (i.e., A Coruña, Lugo, Ourense, and Pontevedra) were taken into account. All the municipalities within each province with more than 10000 inhabitants were included as sampling points. Within each sampling point, subjects were chosen by a random routes procedure to give adequate coverage to the distinct urban streets, neighbourhoods, and rural areas. Once on site, personnel from the project employed a door-to-door recruitment procedure and presented people on an individual basis the possibility to take part in a study dealing with the consumption habits amongst the Galician population. Those who voluntarily accepted to participate and met the inclusion criteria (being between 15 and 65 years of age, currently not under psychopharmacological treatment or psychotherapy, and having no other current impulse control disorder other than compulsive buying) received a paper-version of the questionnaires, and precise information on how to complete them. Additionally, they were given a pre-addressed, postage paid envelope that, after filling in the questionnaires, they were to submit by mail in an approximate period of three weeks. After detailed description of the study to the subjects, written informed consent was obtained, and the confidentiality of the data was guaranteed. The return rate was 41.6%.

The study met and was conducted in compliance with the Helsinki Declaration. Also, it was approved by the Bioethics Committee of the University of Santiago de Compostela.

### Participants

In order to ensure the representativeness of the sample used in this study, the sample was obtained by means of randomly selecting data from the global data file with the assistance of a sociological consulting firm that recommends and advises on the employment of a quota sampling procedure for obtaining a sample that would reproduce the same proportions for different criteria such as gender, mean age, education, and marital status present in the general Galician population, according to the 2011 census, the last completed in this region. In that census, the general population in Galicia was comprised of 48.4% males and 51.6% females. With regards to marital status, 42.9% of inhabitants lived with a partner, and about 57.1% were single, separated, divorced, or widowed. In relation to education, 19% had completed primary education, 63% secondary education, and 18% had an university degree. Conforming to these data, our resulting sample was comprised of 2159 adults (1038 men and 1121 women) with a mean age of 35.4 years (SD = 13.24). Table 1 depicts data corresponding to the frequencies and percentages of the characteristics of the sample according to sex, age ranges, marital status, education, job situation, and perceived social class.

**Table 1:** Sociodemographic characteristics and differences between non-compulsive buyers and compulsive buyers

	**Total sample N = 2159**		**Non-CB N = 2006**		**CB N = 153**		**Comparison Non-CB vs. CB**
	**N**	**%**	**N**	**%**	**N**	**%**	
**Gender**							Χ^2^ = 4.55, p = .033
Male	1038	48.1	978	48.8	60	39.2	
Female	1121	51.9	1028	51.2	93	60.8	
**Age**							Χ^2^ = 25.82, p = .001
15-19	301	13.9	264	13.1	37	24.2	
20-29	629	29.1	579	28.9	50	32.7	
30-39	326	15.1	299	14.9	27	17.6	
40-49	513	23.8	489	24.4	24	15.7	
50-59	345	16	333	16.6	12	7.8	
60-65	45	2.1	42	2.1	3	2	
**Marital status**							Χ^2^ = 2.31, p = .481
Living without a partner	1250	57.9	1134	56.5	116	75.8	
Living with a partner	909	42.1	872	43.5	37	24.2	
**Education**							Χ^2^ = 1.31, p = .52
Primary	436	20.2	408	20.3	28	18.3	
High school	1338	62	1236	61.7	102	66.7	
University	385	17.8	362	18	23	15	
**Job situation**							Χ^2^ = 2.8, p = .43
Without work	957	44.3	863	43	94	61.4	
Working	1202	55.7	1143	57	59	38.6	
**Perceived social class**							Χ^2^ = 2.6, p = .46
Low	44	2	39	1.9	5	3.3	
Middle-low	1214	56.2	1128	56.2	86	56.2	
Middle-high	887	41.2	827	41.3	60	39.2	
High	14	0.6	12	0.6	2	1.3	

Note. Non-CB = Non-compulsive buyers; CB = Compulsive buyers.

### Study variables

#### Compulsive buying

The Spanish translated version of the German Compulsive Buying Scale (GCBS; 13) was employed to evaluate compulsive buying. This questionnaire has 16 items (e.g., “When I have money, I have to spend it”, “Sometimes I buy something that I cannot afford”) which should be answered on a scale from 1 (strongly disagree) to 4 (strongly agree). The total score (range: 16-64) is considered as an indicator of compulsive buying propensity. GCBS has previously demonstrated adequate psychometric properties in other research carried out with Spanish samples [29-31]. In this study, the internal consistency measured using Cronbach’s alpha was .91. For the purpose of the present research, we adopted, in accordance with some previous studies [6,10,3], a cut-off score of two standard deviations above the mean value of the group in GCBS. Given that the mean GCBS score in the total sample was 28.9, and the standard deviation was 7.9, a mark of 45 was taken as the cut-off score for classifying subjects as compulsive buyers.

#### Sociodemographic variables

Participants were initially asked to complete prepared items for assessing sociodemographic characteristics. Data on the variables of sex, age, marital status, education, job situation and perceived social class were collected. In relation to marital status, we distinguished between “living without a partner” (e.g., single, divorced, widowed) or “living with a partner” (e.g., partnership, married). Education was considered in three categories according to the highest level attained: primary, high school, and university. With respect to employment status, two categories were recognized: without work *vs.* working. Finally, as for perceived social class, four categories were included: low, middle-low, middle-high, and high.

#### Psychological distress

This construct was assessed using the anxiety, depression, obsession-compulsion, somatization, and hostility subscales pertaining to the Spanish version [32] of the Symptom Checklist-90-R (SCL-90-R) [33] which provides a measure of psychological symptoms experienced over the month prior to data collection. This instrument has shown adequate psychometric properties in general samples [34] and clinical groups [35] in Spain, with good internal consistency and adequate one-week test-retest correlations. Evidence of concurrent, predictive and discriminant validity has been obtained [35]; moreover, the factor structure of this measure has also been validated [36]. Specifically, it includes ten items related to anxiety (e.g., “Feeling fearful”, “Feeling tense or keyed up”), thirteen items regarding depression (e.g., “Crying easily”, “Blaming self for things”), ten statements evaluating obsession-compulsion (e.g., “Do things slowly to ensure correctness”, “Trouble concentrating”), twelve items assessing somatization (e.g., “Pains in lower back”, “Headaches”), and six statements measuring hostility (e.g., “Easily annoyed or irritated”, “Getting into frequent arguments”). Items are responded to on a scale ranging from 0 (never) to 3 (very often). Items scores are summed to generate a total punctuation on anxiety (range: 0-30), depression (range: 0-39), obsession-compulsion (range: 0-30), somatization (range: 0-36), and hostility (range: 0-18). In this sample, internal consistency indices based on Cronbach’s alpha ranged from .80 for hostility to .91 for anxiety.

#### Coping strategies

The Spanish version [37] of the Coping Strategies Inventory (CSI) [38] was employed to evaluate eight coping strategies, with four of them being active coping strategies: problem solving (e.g., “I made a plan of action and followed it”, “I worked on solving the problems in the situation”), cognitive restructuring (e.g., “I convinced myself that things aren’t quite as bad as they seem”, “I organized the way I looked at the situation so things didn’t look so bad”), express emotions (e.g., “I let my emotions out”, “I got in touch with my feelings and just let them go”), social support (e.g., “I found somebody who was a great listener”, “I talked to someone about how I was feeling”); and four being passive-avoidance coping strategies: problem avoidance (e.g., “I went along as if nothing was happening”, “I avoided thinking/doing anything about the situation”), wishful thinking (e.g., “I wished that the situation would go away or somehow be over with”, “I hoped a miracle would happen”), self-criticism (e.g., “I criticized myself for what happened”, “I blamed myself”), social withdrawal (e.g., “I spent more time alone”, “I avoided being with people”). This measure is comprised of 40 items – five for each coping strategy – that should be answered on a scale of frequency ranging from 1 (never used) to 5 (always used). Adequate psychometric properties have been previously obtained with Spanish samples [39,40]. In the current study, Cronbach’s alpha values ranged from .75 for social withdrawal to .92 for problem solving.

### Statistical analyses

The prevalence of compulsive buying was initially estimated. Accordingly, participants were classified into two groups: compulsive buyers and non-compulsive buyers. Comparisons among these groups in relation to sociodemographic indicators, psychological symptoms, and coping strategies were established using chi-square tests for categorical variables and Anova for continuous determinants. In order to clarify which of these determinants constituted significant predictors of compulsive buying, the variables that were significantly related to this phenomenon at level p < 0.05 in the univariate analyses were then included in a multivariate logistic regression analysis (Enter method). The odds ratio (OR) and the 95% confidence intervals (CI) of the ORs were calculated, with the Wald statistic being used to determine significance of predictors. All statistical analyses were conducted using IBM-PASW Statistics software, version 20.0.

## Results

The prevalence of compulsive buying in the representative Galician population sample was estimated to be 7.1%. Comparisons among compulsive buyers and non-compulsive buyers in relation to sociodemographic determinants (see Table 1) revealed significant prevalence differences by gender, with females showing a higher compulsive buying prevalence than males (8.3% and 5.9%, X^2^ = 4.551, p = .033). Moreover, it was confirmed that women obtained significantly higher scores in GCBS than men (Mean = 30.96, SD = 7.61, and Mean = 27.15, SD = 8.02, t = -11.32, p = .001). Statistically significant differences were also detected with respect to age, with compulsive buyers being significantly younger than the comparison group (Mean = 30.3, SD = 12.87, and Mean = 35.81, SD = 13.19, F_1,2158_ = 24.6, p = .001). In a more in depth analysis focused on distinct age groups, differences between the groups were confirmed again (X^2^ = 25.82, p = .001), with the highest percentage of compulsive buyers corresponding to the age range between 20 and 29 years. As for the remaining demographic determinants examined, comparison results showed that the two groups did not differ at statistically significant levels in relation to marital status, education, job situation, and perceived social class.

In exploring the differences between compulsive buyers and non-compulsive buyers in relation to the distinct dimensions of psychological distress, the results of Anova (Table 2) confirmed that these groups differ significantly in each and every one of the symptoms examined. Specifically, compulsive buyers obtained significantly higher scores than the comparison group with respect to the symptoms of anxiety, depression, obsession-compulsion, somatization, and hostility, with the largest significant differences corresponding to, in this order, obsession-compulsion, depression, and anxiety (F-values ranging from 494.11 to 380.36, p = .001).

**Table 2:** Symptoms and differences between non-compulsive buyers and compulsive buyers

	**Total sample N = 2159**		**Non-CB N = 2006**		**CB N = 153**		**Comparison Non-CB vs. CB**
	**Mean**	**SD**	**Mean**	**SD**	**Mean**	**SD**	
Anxiety	6.18	4.66	5.69	4.1	12.75	6.31	F = 380.36, p = .001
Depression	10.07	6.7	9.35	5.97	19.69	8.26	F = 396.22, p = .001
Obsession-compulsion	9.13	5.36	8.49	4.47	17.6	8.29	F = 494.11, p = .001
Somatization	8.3	5.23	8.04	5.09	11.85	5.73	F = 77.17, p = .001
Hostility	3.2	2.85	3.04	2.74	5.23	3.44	F = 86.21, p = .001

Findings obtained from the comparison among the groups in relation to coping (Table 3) revealed that all the coping strategies considered (except express emotions and social support) established statistically significant differences. More precisely, participants with compulsive buying scored significantly higher on all the passive-avoidance coping strategies of problem avoidance, wishful thinking, self-criticism, and social withdrawal, and significantly lower on the active-focused on the problem strategies of problem solving and cognitive restructuring.

**Table 3:** Coping strategies and differences between non-compulsive buyers and compulsive buyers

	**Total sample N = 2159**		**Non-CB N = 2006**		**CB N = 153**		**Comparison Non-CB vs. CB**
	**Mean**	**SD**	**Mean**	**SD**	**Mean**	**SD**	
Problem solving	15.18	3.43	15.39	3.27	12.33	4.15	F = 117.1, p = .001
Cognitive restructuring	15.64	3.37	15.77	3.22	13.93	4.69	F = 42.65, p = .001
Express emotions	13.1	3.77	13.1	3.55	13.11	5.94	F = .001, p = .974
Social support	15.09	4.49	15.1	4.38	14.84	5.78	F = .502, p = .479
Problem avoidance	12.73	3.49	12.41	3.2	16.93	4.37	F = 263.35, p = .001
Wishful thinking	13.65	3.94	13.29	3.69	18.45	4.1	F = 270.5, p = .001
Self-criticism	13.06	3.81	12.78	3.58	16.73	4.83	F = 161.57, p = .001
Social withdrawal	11.97	3.19	11.84	3.1	13.7	3.74	F = 48.87, p = .001

Lastly, in keeping with the goal of examining the role of sociodemographic determinants, psychological symptoms, and coping strategies as possible risk or protection factors for the development of the compulsive buying phenomenon, a multivariate logistic regression analysis was conducted which included the compulsive buying status (0 = Non-CB, 1 = CB) as the criterion variable, and the variables which in the univariate analyses of variance allowed to differentiate among compulsive buyers and non-compulsive buyers at significant levels (namely, gender, age, anxiety, depression, obsession-compulsion, somatization, hostility, problem solving, cognitive restructuring, problem avoidance, wishful thinking, self-criticism, and social withdrawal) as predictors. Table 4 exhibits the regression analysis results, which confirmed that almost all of the variables considered were significantly associated with compulsive buying, with somatization, hostility and the coping strategy social withdrawal being the only exceptions.

**Table 4:** Results of the logistic regression analysis with compulsive buying as dependent variable

	**B**	**S.E.**	**Wald**	***p***	**OR**	**95% CI**
Gender (male = 0, female = 1)	.121	.031	3.75	.044	1.19	1.012-1.761
Age	-.024	.009	6.98	.008	.977	.96-.994
Anxiety	.064	.033	3.676	.045	1.066	1.011-1.138
Depression	.067	.023	8.073	.004	1.069	1.021-1.119
Obsession-compulsion	.124	.029	18.781	.001	1.132	1.07-1.197
Somatization	-.041	.027	2.396	.122	.959	.91-1.011
Hostility	.011	.038	.09	.765	1.012	.938-1.09
Problem solving	-.179	.04	20.544	.001	.836	.774-.903
Cognitive restructuring	-.101	.038	13.858	.009	.895	.813-.97
Problem avoidance	.189	.037	25.879	.001	1.208	1.123-1.299
Wishful thinking	.068	.031	4.231	.04	1.064	1.013-1.144
Self-criticism	.072	.032	4.905	.027	1.074	1.008-1.144
Social withdrawal	-.036	.037	.938	.333	.964	.896-1.038

Note. Nagelkerke’s R^2^ = 0.466.

Our findings indicated that when the sociodemographic, psychological symptoms, and coping strategies related to compulsive buying at the univariate level were taken together, they significantly predicted whether participants belong to the compulsive buyers or the non-compulsive buyers groups (X^2^ = 440.26, df = 13, p < .001). Moreover, the Nagelkerke R^2^ was .466, suggesting that a large amount of variance of compulsive buying was explained by the predictor variables in the model. Additionally, the logistic regression analysis results showed that being female, the symptoms of anxiety, depression, and obsession-compulsion, and the passive-avoidance coping strategies of problem avoidance, wishful thinking, and self-criticism increased the risk for developing compulsive buying. By contrast, the determinants that were found to act as protection factors against this phenomenon were increased age and employment of the active-focused on the problem coping strategies of problem solving and cognitive restructuring.

## Discussion

This paper aimed to investigate compulsive buying prevalence, and the role of sociodemographic determinants, psychological distress, and coping strategies in this phenomenon in a representative sample of the Galician population (Spain). Findings from the current study suggest that approximately seven of every one hundred individuals in this region present the compulsive buying phenomenon. This group differed from the remaining participants with respect to sex and age. Additionally, they presented significantly higher levels in all the symptoms examined as well as in relation to the employment of passive-avoidance coping strategies. Notwithstanding, individuals with compulsive buying showed significantly lower scores with respect to the use of the active-focused on the problem coping strategies of problem solving and cognitive restructuring. Finally, our results demonstrated that, among the wide variety of variables examined, the determinants that best delineated differences between compulsive buyers and non-compulsive buyers were gender, age, anxiety, depression, obsession-compulsion, problem solving, cognitive restructuring, problem avoidance, wishful thinking, and self-criticism.

In a more exhaustive analysis of these findings, and as it regards the first objective in this study which consisted in the estimation of the prevalence of compulsive buying among the Galician general population, the aforementioned percentage of 7.1% was obtained. This figure is similar to the prevalence rates recently confirmed in some studies with representative samples from European countries, including Germany [6,10], and Denmark [11], and also with the figures detected in other parts of the world such as United States [9].

Examination of the demographic makeup of compulsive buyers relative to non-compulsive buyers (the second aim of this research) revealed that both groups differed significantly with respect to sex and age. Specifically, the compulsive buyers group was comprised of significantly higher percentages of women and younger people. Regarding gender, it should be noted that, although some research has reported finding no statistically significant differences [9,10], our results are more in line with other studies [11,41] which, involving general population samples, not only have detected the existence of significantly higher percentages of women among compulsive buyers, but have also confirmed significantly higher scores in the compulsive buying measures in females as well. Among the possible reasons why women would be more prone to compulsive buying than men, some authors [13] have pointed to the cultural and socialization differential patterns between genders, namely, that in Western culture, women do most of the shopping and spend more time than men engaged in this activity, and, subsequently, it has been surmised that this simple fact of being more exposed to this activity might in itself make females more vulnerable to the compulsive buying phenomenon. The current findings on age also concur with a large body of literature showing that individuals with compulsive buying are significantly younger than people who do not exhibit this behavioural problem [9-11]. More specifically, our results indicated that approximately a third of the compulsive buyers identified in the Galician sample fell within the 20-29 age range. This matches with the findings by Mueller et al. [10] who, taking into account a large German population sample, confirmed that participants between 25 and 34 years of age presented the highest propensity for compulsive buying. This negative link between age and compulsive buying has been repeatedly confirmed [42-44]. In this regard, the presence of third variables, such as the high endorsement of materialistic values among young people, which has been shown to be an effective mediator of the effect of age on compulsive buying [45], jointly with the craving for new experiences and the marked need to reaffirm self identity [46], are some of the explanations which have been reflected in literature. Our results also suggested that the demographic determinants of marital status, education, job situation, and perceived social class did not establish significant differences between those with compulsive buying and the other participants. These findings are in line with the conclusions recently presented by some authors in the compulsive buying field [47] who have underscored, on the one hand, a higher propensity for compulsive buying among women and younger people and, on the other, the absence of a risk profile for this phenomenon on the basis of other demographic characteristics.

In accordance with the third objective in the present study, differences among compulsive buyers and non-compulsive buyers in relation to the dimensions of psychological distress of anxiety, depression, obsession-compulsion, somatization, and hostility were also explored. Our results demonstrated that individuals with compulsive buying experience a significantly higher frequency in all the symptoms analyzed, with the largest differences corresponding to obsession-compulsion. This finding fits with those obtained in some previous studies in the compulsive buying area which have also confirmed the presence of high scores in obsession-compulsion among compulsive buyers [23] as well as the existence of significant differences between compulsive buyers and non-compulsive buyers for this type of symptoms [21]. Indirect empirical evidence along these lines can also be gleaned from literature in similar areas like impulse control disorders, where the prominent role of obsessive thoughts as a risk factor for anorexia and bulimia has been highlighted [48]. As for the symptoms of anxiety, depression, and hostility, it should be noted that the evidence obtained in this study concurs with that derived from some studies demonstrating high frequencies of the experience of feelings of sadness, anxiety, and anger/hostility before compulsive buying episodes on the part of compulsive buyers [17,24]. In the same vein, the findings from a recent study on the differences between three groups with low, medium, and high propensity for compulsive buying on the basis of the Big Five personality traits and their thirty facets provided further confirmation [49]. That particular study demonstrated that participants in the group with a high vulnerability to compulsive buying presented significantly higher scores in the anxiety, depression, and angry hostility facets. In view of this evidence, and taking into account the conclusions from other recent studies [50] suggesting the existence of a close relationship between the experience of anxiety and depression, on the one hand, and physical malaise, on the other, a tentative explanation for our findings could be postulated. To that effect, and aware that this is the first study which has detected significantly higher levels of physical upsets (gastrointestinal, breathing, and muscular upsets, for instance) among compulsive buyers, it seems reasonable to think that somatization, together with anxiety, depression, obsession-compulsion, and hostility, forms part of a setting of marked physical and psychological malaise leading to the engagement in compulsive buying episodes.

Analysis of the differences between compulsive buyers and the remaining participants in relation to the coping strategies (fourth objective) highlighted that individuals with compulsive buying present a coping pattern characterized, on the one hand, by a significantly higher frequency of employment of each and every one of the passive-avoidance coping strategies (specifically, problem avoidance, wishful thinking, self-criticism, and social withdrawal) and, on the other, by a lower frequency of use of the active-focused on the problem coping strategies of problem solving and cognitive restructuring. These findings revealed the prominent role of a maladaptive coping style characterized by the combination of passive-avoidance coping strategies and the lack of, or the inadequate use of, active-focused on the problem strategies in the compulsive buying phenomenon, and are in agreement with prior data from research developed in similar areas including impulse control disorders like pathological gambling [26], and eating disorders [27]. For instance, Jáuregui et al. [27] confirmed that patients with anorexia and bulimia obtained, relative to those participants without eating disorders, significantly higher scores in the passive coping strategies of self-criticism, social withdrawal, and wishful thinking.

In accord with the last and major aim of this paper entailing the determination of which variables belonging to the different sets analyzed (demographic, psychological distress, and coping strategies) best delineate differences among compulsive buyers and non-compulsive buyers in the Galician sample considered, our results confirmed that gender (namely, being a woman), the symptoms of anxiety, depression, and obsession-compulsion, and the passive-avoidance coping strategies of problem avoidance, wishful thinking, and self-criticism, all emerged as risk factors in relation to compulsive buying, increasing the likelihood of participants to be classified as compulsive buyers. On the contrary, increased age and the employment of problem solving and cognitive restructuring as coping strategies constitute, in view of our findings, protection factors against the development of the phenomenon under study. Unfortunately, more in depth discussion on these findings is limited by the scarcity of previous studies that have considered the cited sets of variables jointly, with available evidence fundamentally making reference only to demographic determinants. In this regard, it should be noted that our results concur with those obtained in some earlier studies that have also commenced with the identification of participants with compulsive buying in representative general population samples in order to determine who was at risk for becoming a compulsive buyer [9,10]. Thus, Mueller et al. [10] confirmed that young people and subjects with depression symptoms presented the highest propensity for compulsive buying in a representative sample from Germany. Complementary evidence in regards to the relationship between younger aged people and compulsive buying detected in the current study has been also obtained by Koran et al. [9] in the United States; in particular, these authors confirmed that those of younger age presented a higher vulnerability to this behavioural problem. However, as has been said, this study also intended to illuminate the role of other factors that include psychological distress and coping styles in compulsive buying, and, with this in mind, the evidence obtained regarding obsession-compulsion, anxiety, and depression symptoms being significantly associated with the membership to the compulsive buying group is in agreement with some of the explanatory hypothesis presented over the last few years in relation to the possible motivations for this problem. Thus, the possibility that buying may act as a way of self medication providing short-term relief not only for depressive and anxiety symptomatology, but also for the marked tension linked to obsessive rumination has been considered [5,17,51]. It also seems reasonable to think that the cited maladaptive coping style detected among those participants showing compulsive buying patterns (comprised of the overutilization of passive-avoidance coping strategies and the misuse of the active-focused in problem coping strategies), far from facilitating the setting in motion of the resources and skills necessary for the appropriate management of stressful situations and symptoms, would actually increase, as our results have demonstrated, one’s likelihood of becoming a compulsive buyer. In fact, some authors have suggested that the act of buying itself might function as a coping mechanism for dealing with problems and negative feelings, or serve as a way to escape from reality and block out obsessive thoughts [47]. This thesis seems particularly suitable for the framework of this study given the economic situation of financial crisis that Spain is currently going through. Within this background, painted by a blurred present where known coordinates have broken down and the future is seen as uncertain and bleak, it may be reasoned that buying in and of itself might function as a compensatory mechanism in addressing the in-depth malaise derived from the economic reality of the country.

The current study presents a number of strengths, including its relatively large sample size and representativeness as it pertains to the Galician population. It is also worth noting that to the best of our knowledge this research is the first of its kind, echoing the multifactorial etiology of compulsive buying, and establishing a risk profile for this phenomenon based on distinct sets of variables: sociodemographic, psychological distress, and coping strategies. In this direction, our findings revealed that females, younger people, subjects experiencing symptoms of obsession-compulsion, depression, anxiety, and/or people who tend to employ the passive-avoidance coping strategies (specifically, problem avoidance, wishful thinking, and self-criticism) to a greater extent and the active-focused on the problem strategies of problem solving and cognitive restructuring to a lesser extent, all showed higher propensities for compulsive buying.

Some practical and clinical implications could be derived in light of these results. For instance, the design and implementation of comprehensive programs which include specific components aimed at the relief of psychological distress and the promotion of the active coping strategies that our study has identified as protection factors against compulsive buying (i.e., problem solving and cognitive restructuring) seem to be advisable. Adaptation and employment of specific components taken from cognitive-behavioural programs which have previously demonstrated their effectiveness in the compulsive buying treatment [52-54] should also be considered. To be precise, they include components designed to manage obsessive thoughts and compulsive buying patterns (e.g., exposition with response prevention), alleviate anxiety and depressive symptoms, and promote adaptive stress management and the employment of problem solving strategies [55]. In addition, and given that our results confirmed that younger people showed a greater vulnerability to compulsive buying, the prevention of unhealthy consumer behaviours should probably be put on the agenda in high-school and university health programs.

Notwithstanding, there are a number of limitations and issues that warrant attention and that should be kept in mind for future research. Firstly, compulsive buyers were exclusively identified by means of a screening instrument, resulting in a possible over- or underestimation in the prevalence rate; hence, advocating future studies to combine both screening instruments and validated clinical interviews with the aim of obtaining greater precision in the compulsive buying diagnostic seems appropriate. Secondly, due to the fact that all the variables were assessed by self-report measures, socially desirable responses may have been given. Thirdly, potential concerns related to generalizability could be solved by means of carrying out additional studies with populations from other countries and cultures. Finally, inferences about causation between variables cannot be raised because of the cross-sectional nature of data.

## Conclusions

The results of this study add to the understanding of compulsive buying and suggest the implication of a variety of determinants of distinct nature in this phenomenon. The estimated prevalence of compulsive buying among the Galician general population is 7.1%. In exploring differences between compulsive buyers and non-compulsive buyers based on sociodemographic determinants, psychological symptoms and coping styles, the fact that women and young people show a significantly higher vulnerability to this behavioural problem was confirmed. Additionally, compulsive buyers present, relative to non-compulsive buyers, significantly higher levels in the symptoms of obsession-compulsion, depression, anxiety, hostility, and somatization. As regards to coping, individuals with compulsive buying show significantly higher scores in the passive-avoidance coping strategies of problem avoidance, wishful thinking, self-criticism, and social withdrawal, and significantly lower scores on the active-focused on the problem coping strategies of problem solving and cognitive restructuring. Finally, in establishing a prototypical risk profile for compulsive buying in this region, our findings demonstrate that gender (being female), experiencing obsessive-compulsive, depression, and anxiety symptoms, and the employment of the passive-avoidance coping strategies of problem avoidance, wishful thinking, and self-criticism constitute risk factors in relation to the phenomenon under study, whilst increased age and the use of the active-focused on the problem coping strategies of problem solving and cognitive restructuring are protection factors against compulsive buying. The detection of these risk and protection factors will contribute not only to the better identification of people with a high vulnerability to this problem, but also to the tailoring of specific prevention and treatment components, according to the characteristics of each subject, which would ultimately increase the effectiveness of compulsive buying intervention programmes.

## Competing interests

The authors declare that they have no competing interests.

## Authors’ contributions

JMOL designed the study, conducted statistical analyses, and interpreted the results. EV collected the data and made literature reviews. Both authors drafted the manuscript and approved the final version.

## Authors’ information

^1^JMOL is Titular Professor in the Department of Clinical Psychology and Psychobiology, University of Santiago de Compostela, Spain. ^2^EV is a researcher of the group “Problemas sociales: Análisis, evaluación e intervención” at the University of Santiago de Compostela, Spain.

## Pre-publication history

The pre-publication history for this paper can be accessed here:

http://www.biomedcentral.com/1471-244X/14/101/prepub
